# Examination of Urine in Disease

**Published:** 1863-08

**Authors:** 


### EXAMINATION OF URINE IN DISEASE.

M. Bonchardat has arranged the necessary agents for such an examination, so as to make the matter easy. The principal agents employed are heat, nitric acid, tannin, lime, and ioduretted solution of the iodide of potassium. 1—*Heat.* A little above 100°; the urine becoming turbid, will separate albumen in the form of clots or flakes. But it must not be forgotten that every specimen of urine which becomes turbid, under the agency of heat, is not necessarily albuminous; hence we must employ, 2—*Nitric Acid.* Add some of this acid carefully to the urine. If albumen be present flakes will be deposited. An excess of acid will dissolve the flakes. Any urine that becomes turbid under the influence of heat, and renders a precipitate under the action of nitric acid, contains, beyond all doubt, albumen. 3—*Tannin.* A solution is made by adding two hundred *grammes* of water to ten *grammes* of tannin, and ten *grammes* of ether are then added to preserve the solution. The action of this re-agent cannot be relied upon, since the solution of tannin will produce an abundant precipitate, if soup, rich in gelatine, have been taken shortly before the analysis. 4—*Lime.* This detects admirably the sugar of diabetes. Fifty *grammes* of urine, and two *grammes* of lime, being boiled together in an assay bottle, will give the color of caramel, more or less dark, according to the quantity of sugar present. The quick-lime is slaked with water, and then introduced into a flask which has a tight cork. If fifty *grammes* of urine are not colored when boiled with two *grammes* of lime, then two *grammes* more should be added. After the mixture is boiled again, if no color appear, it is well to test the lime. For this purpose, a half teaspoonful of starch sugar is poured into the flask. On boiling, if the urine should then be freely colored, we have proof that the lime had been properly calcined, and that the urine contained no sugar independent of the starch-sugar which had been added. 5—*Ioduretted Solution of Iodide of Potassium.* This is prepared by dissolving one part of iodine and one part of iodide of potassium in fifty parts of water. This solution will communicate a chesnut brown color to urine containing sulphate of quinine or any other alkaloid likely to be administered in therapeutics.—Jour. de Chim. Med. and Amer. Med. Monthly.—[*Dublin Medical Press, Feb.* 18, 1863, *p.* 59.

